# Bubble CPAP and oxygen for child pneumonia care in Malawi: a CPAP IMPACT time motion study

**DOI:** 10.1186/s12913-019-4364-y

**Published:** 2019-07-31

**Authors:** Kristen L. Sessions, Tisungane Mvalo, Davie Kondowe, Donnie Makonokaya, Mina C. Hosseinipour, Alfred Chalira, Norman Lufesi, Michelle Eckerle, Andrew G. Smith, Eric D. McCollum

**Affiliations:** 1 Mayo Clinic School of Medicine, 200 1st Street SW, Rochester, MN USA; 2 University of North Carolina Project Malawi, Tidziwe Centre, 100 Mzimba Road, Lilongwe, Malawi; 3 Community Health Science Unit, Private Bag, 65 Lilongwe, Malawi; 4 University of Cincinnati College of Medicine, 3333 Burnet Ave, Cincinnati, OH 45229 USA; 5 University of Utah, P.O. Box 581289, Salt Lake City, UT 84158 USA; 6 Johns Hopkins School of Medicine, Rubenstein Child Health Building, #3150, 200 North Wolfe Street, Baltimore, MD 21287 USA; 7 Northwestern University, Lurie Children’s Hospital, 225 E, Chicago, IL 60611 USA

**Keywords:** Time motion study, Bubble continuous positive airway pressure, Child pneumonia, Sub-Saharan Africa, Oxygen

## Abstract

**Background:**

In some low-resource settings bubble continuous positive airway pressure (bCPAP) is increasingly used to treat children with pneumonia. However, the time required for healthcare workers (HCWs) to administer bCPAP is unknown and may have implementation implications.

This study aims to compare HCW time spent administering bCPAP and low-flow nasal oxygen care at a district hospital in Malawi during CPAP IMPACT (Improving Mortality for Pneumonia in African Children Trial).

**Methods:**

Eligible participants were 1–59 months old with WHO-defined severe pneumonia and HIV-infection, HIV-exposure, severe malnutrition, or hypoxemia and were randomized to either bCPAP or oxygen. We used time motion techniques to observe hospital care in four hour blocks during treatment initiation or follow up (maintenance). HCW mean time per patient at the bedside over the observation period was calculated by study arm.

**Results:**

Overall, bCPAP required an average of 34.71 min per patient more than low-flow nasal oxygen to initiate (bCPAP, 118.18 min (standard deviation (SD) 42.73 min); oxygen, 83.47 min (SD, 20.18 min), *p* < 0.01). During initiation, HCWs spent, on average, 12.45 min longer per patient setting up bCPAP equipment (*p* < 0.01) and 11.13 min longer per patient setting up the bCPAP nasal interface (*p* < 0.01), compared to oxygen equipment and nasal cannula set-up. During maintenance care, HCWs spent longer on average per patient adjusting bCPAP, compared to oxygen equipment (bCPAP 4.57 min (SD, 4.78 min); oxygen, 1.52 min (SD, 2.50 min), *p* = 0.03).

**Conclusion:**

Effective bCPAP implementation in low-resource settings will likely create additional HCW burden relative to usual pneumonia care with oxygen.

**Trial registration:**

Clinicaltrials.gov NCT02484183, June 29, 2015.

**Electronic supplementary material:**

The online version of this article (10.1186/s12913-019-4364-y) contains supplementary material, which is available to authorized users.

## Background

Pneumonia is a leading cause of under-five mortality worldwide, responsible for an estimated 704,000 child deaths annually [1]. HIV infection, HIV exposure, severe acute malnutrition (SAM), and severe hypoxemia (SpO_2_ < 90%) are independent risk factors for mortality among children with pneumonia [2–8]. Current standard of care for children with World Health Organization (WHO)-defined severe pneumonia includes hospitalization, parenteral antibiotics, and administration of low-flow nasal oxygen; however, advanced respiratory treatment modalities may further decrease mortality [9]. In low resource sub-Saharan African countries such as Malawi, the majority of children hospitalized for pneumonia are treated within the general pediatric wards at the district hospital level where intensive care services are not available [10]. Therefore, to maximize the public health impact in most African settings, advanced treatment modalities must be effective and feasible in a district hospital setting.

Bubble continuous positive airway pressure (bCPAP) is a relatively simple and inexpensive respiratory support modality that delivers constant positive end expiratory pressure (PEEP) to re-recruit and stabilize collapsed lung alveoli, widen narrowed airways, and optimize ventilation perfusion matching to reduce work of breathing and improve oxygenation in children with severe lower respiratory disease [11]. In bCPAP, a closed respiratory circuit generates PEEP by submerging the end of the expiratory tubing into a bottle of sterile water. The depth of the tube in the water correlates with the amount of PEEP generated. bCPAP is used in many developed countries to treat neonates with severe respiratory distress and can decrease morbidity and mortality among these patients [12–14]. There is evidence that bCPAP can also be administered safely and effectively to treat neonates in low-resource settings [15–18]. bCPAP efficacy for treating older children with pneumonia and other lower respiratory diseases like pneumonia has not been as well studied. In one randomized control trial in Bangladesh, bCPAP reduced the risk of mortality among children with severe hypoxemic pneumonia [19]. Another trial in Ghana found no mortality benefit from conventional CPAP use among children with severe respiratory distress at the district hospital level but did find lower mortality among children < 12 months old in a secondary analysis [20].

While bCPAP may reduce mortality in children with severe pneumonia, effective administration may require more intensive nursing care [21]. Scale up in resource-limited countries, like Malawi, may therefore require substantial physical and human resources to meet expected needs [22]. Before bCPAP is widely implemented, it is critical to objectively understand whether additional healthcare worker time is required to administer effective care, compared to current standards, and what the implications of increased care demands from bCPAP may have on district hospital healthcare workers. Understanding human resource needs can help inform larger cost-benefit analysis as well as inform workflow patterns for implementation of bCPAP in new settings.

Continuous observation time motion studies (TMS) are considered the gold standard in measuring clinical workflow [23–27]. Using an external observer to continuously record the time taken to complete unique activities, TMS are used to study efficacy and efficiency of health care administration [23, 28]. TMS has been widely used to observe physician and nursing workflow in both developed and developing countries [29–35]. There are no TMS studies evaluating bCPAP in low-resource settings like Malawi.

In this study we addressed this evidence gap by conducting a continuous observation TMS documenting the amount of time required to administer bCPAP, compared to low-flow nasal oxygen, among children with severe pneumonia in a district hospital in Malawi. This analysis is intended to inform larger cost-benefit analyses and implementation science projects seeking to better implement bCPAP in low resource settings. This study took place as part of a randomized control trial called CPAP IMPACT (Improving Mortality for Pneumonia in African Children Trial) [36]. CPAP IMPACT investigated whether bCPAP confers a mortality benefit, compared to low-flow nasal oxygen, among high risk children 1–59 months of age with WHO-defined severe pneumonia.

## Methods

### Study site and sample

This study was conducted in the pediatric ward at Salima District Hospital (SDH), Salima, Malawi. SDH is a 250 bed government facility that admits approximately 7,000 children annually. SDH is located in the central region of rural Malawi, about 100 km from the capital of Malawi, Lilongwe.

Patients were enrolled in the study as part of CPAP IMPACT. Patients eligible for the study were 1–59 months of age with WHO-defined severe pneumonia and either HIV-infection, HIV-exposure, severe acute malnutrition, or hypoxemia (an oxygen saturation < 90% as determined by pulse oximetry measurement). All patients enrolled into CPAP IMPACT were eligible for the TMS. Children randomized to the bCPAP group received bCPAP through either a nasal mask or nasal prongs. Patients received care per study protocol that included twice-daily clinical reviews by a clinician or nurse, vital signs every six hours, and as needed nursing care. This study was approved by the National Health Science Research Committee of Malawi and the Institutional Review Board of John Hopkin’s University (clinicaltrial.gov NCT02484183).

### Observations

Patients were continuously observed either for the first four hours of trial initiation, regardless of the time of day, or for four hours the morning after enrolment, beginning at 08:00 am, which was considered representative of maintenance care. Observations for the initiation period began at time of randomization to bCPAP or low-flow nasal oxygen, which followed written informed consent. Participants were randomized to bCPAP or oxygen and we planned to observe a total of 68 observation periods, equally distributed between initiation and maintenance as well as intervention and control patients. Patients enrolled in bCPAP could be observed during treatment initiation, maintenance, or both time periods dependent on observer availability. All activities by health care workers that occurred at the bedside were recorded. For this study, healthcare workers included non-physician clinicians called clinical officers, nurses, vital sign assistants, and HIV counselors. Vital sign assistants are lay personnel trained to collect vital signs and assist nurses with other basic care duties. Clinical officers and nurses were chosen to work on CPAP-IMPACT based on previous experience with bCPAP and the clinical trial was ongoing for over two years at time of observations.

Activities anticipated to be observed included, but were not limited to, communications with patient and other staff members, physical examinations, trial-specific digital auscultation lung recordings, trial-specific video recordings of breathing patterns, vital signs, blood draws, medication administration, nasal suctioning, and set up, cleaning, adjustments, and trouble shooting of respiratory equipment (Table 1). The final list of observations was finalized during pilot testing. The number of staff members caring for the patient during a given observation period was also recorded.

**Table 1:** Description of Observed Activities

Task	Description
Communication	Verbal communication with colleague or caregiver while not completing any other activity
Documentation	Writing in chart or filling of forms including paper and tablet documentation
Physical Examination	Physical examination, not including vitals
Vital signs	Counting respirations and measuring oxygen saturation, blood pressure, and temperature
Digital auscultation lung sound recording	Digital recording of patient lung sounds
Video recording of breathing	Video recording of respiration pattern
Blood draw	Blood draw for point of care or laboratory based tests including malaria rapid test, malaria smear, capillary blood gas, hemoglobin test, HIV rapid test, blood glucose
Medication administration	Setting up and administering medications
Feeding	Administering feeding including oral and nasogastric feeds and insertion of a nasogastric tube
Suctioning	Nasal or oral suctioning
Adjustment of equipment	Set up and adjustment of all parts of the bCPAP or oxygen equipment except the nasal interface
Adjustment of nasal interface	Set up and adjustment of mask, head gear, or nasal prongs including ensuring tight seal of mask around patient’s nose
Not at bedside	Person not at bedside
Not otherwise specified	Task not otherwise specified

### Study design and data collection tool

This study was designed using a previously developed TMS checklist [37]. Observations took place between December 2017 and March 2018 by two trained observers. Each observer was trained on the data collection tool as well as practice sessions during both initiation and maintenance periods. Data was initially collected using TimeCat 3.9™, a comprehensive TMS data collection tool. However, due to inconsistent internet network access at the hospital, most data collection was completed using a paper data collection tool and stopwatch. The start and stop times of each activity, which health care provider was providing care, type of care provided, and descriptive details were recorded for each unique activity that occurred at the bedside. In the event of multitasking by a single provider, the time was assigned to the primary task. In the event of multiple providers simultaneously providing care to the patient, the tasks of each provider were recorded independently. Observers only interacted with staff to clarify specific medical procedures.

### Interobserver reliability assessment (IORA)

In order to ensure observations were consistent between the two observers, the observers underwent IORA prior to data collection activities [38]. IORA was done using the TimeCaT 3.9™ system [39] which assesses agreement in the naming, timing, and sequence of activities and reports, and reports a proportion-kappa (P-K) statistic as a measure of inter-observer agreement. Study data collection was not initiated until P-K was > 0.75, which required a total of 8 h of preparatory observation (Additional file 1: Table S1).

### Statistical analysis

Data was described using descriptive statistics to determine the mean time spent by each provider at the patient bedside in one 4 h period. Analysis was also subdivided by individual task and cadre of medical professional. Frequency of each task and total number of unique tasks per 4 h was used as a measure of task shifting and work flow patterns. bCPAP and oxygen cohorts were compared using a two sided t-test.

## Results

### Demographics

A total of 40 patients (19 bCPAP, 20 low-flow nasal oxygen) were observed for 55 four-hour observation periods. Patients observed had a mean age of 11.35 months (SD 10.48) and 40% were female (Additional file 2: Table S2). There were no differences between patients in the bCPAP and low-flow oxygen arms in terms of age, sex, weight, comorbidities, and disease severity. Children in the low-flow nasal oxygen arm had significantly higher average temperature (bCPAP 37.14, SD 1.31; low-flow nasal oxygen 38.05, SD 1.13, *p* = 0.02). There were also no significant differences in terms of age, sex, weight, and disease severity between children observed in this sub-study and all CPAP-IMPACT participants (Additional file 3: Table S3). Healthcare providers observed included clinical officers, nurses, HIV counselors, and vital sign assistants (Table 2).

**Table 2:** Demographic information of healthcare workers providing care

	*N* = 12
Role, n (%)	
Clinical Officer	2 (16.7)
Nurse	5 (41.7)
HIV Counselor	3 (25.0)
Vital Sign Assistant	1 (8.3)
Years of experience, mean (SD)	6.9 (5.3)

SD indicates standard deviation; HIV, human immunodeficiency virus

### Time at patient bedside

During the first 4 h of care, healthcare workers spent an average of 34.71 min longer, per patient, initiating bCPAP compared to low-flow oxygen (118.18 and 83.47 min respectively) (Table 3). Among both groups, nurses spent the most time at the bedside during treatment initiation.

**Table 3:** Mean time per patient spent by healthcare providers on unique bedside activities over a 240 min initiation period

Task, mean minutes (SD)	bCPAP Initiation (*n* = 12)	Oxygen Initiation (*n* = 11)	*p* value
All	118.18 (42.73)	83.47 (20.18)	0.02*
Healthcare provider cadre			
Clinical officer	27.43 (36.67)	20.62 (20.85)	0.59
Nurse	70.40 (51.70)	48.53 (34.95)	0.25
Vital sign assistant	1.20 (3.15)	1.48 (2.33)	0.86
HIV counselor	19.13 (16.00)	12.85 (20.83)	0.42
Non-trial specific tasks			
All	107.15 (42.22)	73.82 (19.35)	0.03*
Communication	18.98 (22.23)	13.73 (8.12)	0.47
Documentation	37.62 (14.25)	30.77 (11.63)	0.22
Physical Examination	0.85 (1.13)	0.52 (0.82)	0.47
Vital signs	3.77 (2.27)	3.67 (2.25)	0.91
Adjustment of equipment	15.28 (7.27)	2.83 (2.17)	< 0.01*
Adjustment of nasal interface	13.78 (8.93)	2.65 (2.78)	< 0.01*
Suctioning	1.40 (3.22)	1.03 (2.08)	0.73
Blood draw	7.40 (5.15)	9.17 (7.90)	0.54
Medication Administration	0.67 (0.82)	1.83 (1.62)	0.03*
Feeding	2.47 (4.15)	0.05 (0.18)	0.07
Not otherwise specified	4.92 (7.87)	8.23 (6.67)	0.29
Trial-specific tasks			
All	11.02 (2.20)	9.67 (2.52)	0.17
Video recording of breathing	5.33 (1.42)	4.95 (2.35)	0.62
Digital auscultation lung sound recording	5.68 (1.78)	4.70 (1.78)	0.23

bCPAP indicates bubble continuous positive airway pressure; SD, standard deviation; HIV, human immunodeficiency virus

*p < 0.05 considered statistically significant

The two tasks most specific to bCPAP or oxygen therapy were equipment and nasal interface set up and adjustments. Both tasks took significantly longer to perform during initiation of bCPAP therapy, compared to oxygen initiation (Table 3). Healthcare workers spent an additional 12.45 min setting up and adjusting bCPAP equipment, compared to oxygen, (bCPAP 15.28 min, SD 7.27; oxygen 2.83 min, SD 2.17) and an additional 11.13 min setting up and adjusting the bCPAP nasal interface, compared to setting up and adjusting the oxygen nasal cannula (bCPAP 13.78 min, SD 8.93; low-flow nasal oxygen 2.65 min, SD 2.78). All other tasks during treatment initiation were comparable between the bCPAP and oxygen groups except medication administration, which took longer in the oxygen group (bCPAP 0.67 min, SD 0.82; low-flow nasal oxygen 1.83 min, SD 1.62).

During 4 h of maintenance care the morning after treatment initiation, there was no significant difference in the overall time spent at the patient bedside (bCPAP 38.50, SD 34.93; oxygen 26.88, SD 15.50) (Table 4). However, healthcare workers spent significantly longer adjusting bCPAP equipment, compared to low-flow nasal oxygen equipment. Healthcare providers spent a total of 4.8 min longer, over a 4 h period, adjusting the bCPAP equipment and nasal interface than adjusting oxygen equipment and the nasal cannula.

**Table 4:** Mean time per patient spent by healthcare providers on unique bedside activities over a 240 min maintenance period

Task, mean minutes (SD)	bCPAP Maintenance (*n* = 17)	Oxygen Maintenance (*n* = 15)	*p* value
All	38.50 (34.93)	26.88 (15.50)	0.24
Healthcare provider cadre			
Clinical officer	11.18 (9.48)	13.63 (14.48)	0.56
Nurse	21.32 (28.70)	9.17 (7.17)	0.12
Vital sign assistant	2.92 (3.82)	3.88 (8.20)	0.69
HIV counselor	2.08 (5.82)	3.88 (0.65)	0.24
Non-trial specific tasks			
All	38.50 (34.93)	26.88 (15.50)	0.24
Communication	6.72 (9.35)	4.63 (6.15)	0.46
Documentation	14.85 (9.95)	13.97 (8.70)	0.79
Physical Examination	1.67 (4.37)	1.13 (1.00)	0.66
Vital signs	3.78 (4.70)	2.35 (1.47)	0.28
Adjustment of equipment	4.57 (4.87)	1.52 (2.50)	0.04*
Adjustment of nasal interface	2.38 (3.42)	0.63 (1.53)	0.08
Suctioning	0.47 (1.58)	0.30 (0.85)	0.82
Blood draw	0.07 (0.17)	0.05 (0.12)	1.00
Medication Administration	1.43 (2.63)	1.55 (2.32)	0.91
Feeding	0.33 (0.78)	0 (0)	0.11
Not otherwise specified	2.25 (5.43)	0.77 (1.78)	0.31

There were no study specific tasks during the maintenance period

bCPAP indicates bubble continuous positive airway pressure; SD, standard deviation; HIV, human immunodeficiency virus

*p < 0.05 considered statistically significant

### Frequency of events

During initiation of bCPAP, healthcare workers performed, on average, 26.40 more unique tasks than during initiation of oxygen (103.58 and 77.18 respectively) (Table 5). The bCPAP equipment was adjusted on average 5.58 more times than the equipment for oxygen over 4 h (8.58 and 3.00, *p* < 0.001). Additionally, the nasal bCPAP interface was adjusted an average of 9.17 times compared to 2.55 times for the oxygen nasal cannula over the fours of initiation (*p* < 0.001).

**Table 5:** Number of occurrences of each task performed by a healthcare worker at the patient bedside over a 240 min initiation period or a 240 min maintenance period

Task, mean number of occurrences (SD)	bCPAP Initiation (*n* = 12)	Oxygen Initiation (*n* = 11)	*p* value	bCPAP Maintenance (*n* = 17)	Oxygen Maintenance (*n* = 15)	*p* value
All	103.58 (42.29)	77.18 (15.92)	0.07	46.18 (27.47)	30.00 (12.51)	0.04
Non-trial tasks						
All	99.25 (41.56)	72.82 (15.67)	0.06	46.18 (27.47)	30.00 (12.51)	0.04
Communication	16.00 (9.66)	11.27 (4.88)	0.16	5.94 (5.45)	13.60 (3.94)	< 0.01*
Documentation	11.67 (4.68)	12.36 (5.07)	0.74	6.35 (3.33)	5.73 (2.55)	0.56
Physical Exam	1.42 (1.08)	1.09 (1.45)	0.54	1.94 (3.65)	1.60 (1.12)	0.73
Vital signs	2.92 (1.56)	3.64 (2.16)	0.37	2.65 (1.66)	1.87 (0.83)	0.11
Adjustment of equipment	8.58 (2.78)	3.00 (2.10)	< 0.01*	4.47 (3.32)	1.00 (1.20)	< 0.01*
Adjustment of nasal Interface	9.17 (4.88)	2.55 (2.16)	< 0.01*	2.65 (2.87)	0.73 (1.58)	0.03*
Suctioning	0.58 (1.24)	0.55 (1.21)	0.95	0.24 (0.56)	0.20 (0.56)	0.84
Blood draw	3.92 (2.19)	3.73 (1.56)	0.81	0.12 (0.33)	0.13 (0.35)	0.93
Medication Administration	0.83 (0.94)	1.55 (1.29)	0.14	1.12 (1.58)	0.73 (0.80)	0.40
Feeding	0.83 (1.40)	0.09 (0.30)	0.10	0.24 (0.56)	0.00 (0.00)	0.11
Not otherwise specified	6.83 (7.79)	4.91 (2.84)	0.45	1.59 (2.37)	0.67 (1.11)	0.18
Trial-specific tasks						
All	4.33 (1.23)	4.36 (0.50)	0.94	N/A	N/A	N/A
Video recording of breathing	2.17 (0.94)	2.36 (3.73)	0.87	N/A	N/A	N/A
Digital lung auscultation recording	2.17 (0.72)	2.00 (0.45)	0.51	N/A	N/A	N/A

bCPAP indicates bubble continuous positive airway pressure; SD, standard deviation; HIV, human immunodeficiency virus

*p < 0.05 considered statistically significant

N/A = not applicable. There were no study specific tasks performed during the maintenance phase of this study. All study specific tasks occurred during initiation of treatment

Similarly, during maintenance therapy, bCPAP required on average an additional 16.18 unique tasks (bCPAP 46.18, low-flow nasal oxygen 30.00) over 4 h. bCPAP equipment was adjusted an average of 3.47 more times than oxygen equipment and the bCPAP nasal interface was adjusted an additional 1.92 times per 4 h compared to the oxygen nasal cannula. During the maintenance phase, healthcare workers communicated with caregivers and other staff, without simultaneously performing other tasks, on an average of 7.66 more occasions in the oxygen than the bCPAP group (bCPAP 5.94, oxygen 13.60, *p* < 0.01).

## Discussion

There is increasing recognition in healthcare that inefficiencies in workflow can lead to increased human resource demands and costs [23]. This is particularly important in low resource settings where human and financial resources are limited. The potential to disrupt workflow and increase work demands are commonly cited barriers for implementation of new technologies [35, 40]. While new technologies, such as bCPAP, may provide a mortality benefit to children with severe respiratory distress in some settings, little is known about the human resource needs required to support effective bCPAP implementation in resource-limited settings. Our study demonstrates that within the context of a clinical trial designed to be representative of real-world care, bCPAP care takes significantly longer to initiate and maintain, and also requires more frequent visits to the patient bedside, than low-flow nasal oxygen. Over a 4 h period, we found providers administering bCPAP took an average of 35 min longer per patient to set up the equipment and nasal interface and spent an additional 5 min per patient to maintain appropriately functioning equipment. Compared to oxygen, healthcare providers performed 26 additional unique tasks during the first 4 h of care and 16 additional tasks every 4 h after initiation for bCPAP patients. These additional tasks and time represent a substantial increase in work load.

During CPAP IMPACT, the SDH pediatric ward hospitalized an average of 331 total patients per month and an average of 19 patients monthly with WHO-defined severe pneumonia and a high-risk comorbidity. This pediatric ward has, on average, seven clinical staff to administer care, which is an average total provider to total patient ratio of 47:1. If bCPAP initiation takes 35 additional minutes per patient over a 4 h period and maintenance bCPAP care takes an additional 5 min per patient over 4 h during three days of hospitalization, bCPAP administration to all hospitalized children with severe pneumonia and a comorbidity could add as much as 33 additional hours per month of care. During peak pneumonia season, the number of patients meeting these criteria can increase to 45 patients per month, which would represent an additional 78 work hours per month on average. Additionally, SDH treats an average of 50 patients per month with WHO-defined severe pneumonia without HIV, malnutrition, or hypoxemia, and up to an average of 94 patients monthly during peak pneumonia season. If all patients with severe pneumonia, regardless of comorbidity, received bCPAP instead of low-flow nasal oxygen therapy health care workers could face an additional 164 h of work per month during peak pneumonia season.

During both the initiation and maintenance phases, the majority of extra time and tasks were devoted to adjustment of the equipment and nasal interface. bCPAP equipment, compared to a nasal cannula used for oxygen delivery, has more parts and requires a complete seal against the patient’s face to be effective. The complete seal is a critical component of successfully delivering bCPAP treatment as leaks will reduce PEEP. Providers must also ensure that the bCAP water reservoir has sufficient fluid levels and appropriate bubbling is occurring, and that there is no residual fluid collecting within bCPAP tubing. Effective bCPAP delivery requires frequent attention all of these components; this suggests that there may be need for further innovation within the bCPAP system to improve the feasibility of maintaining a sealed nasal interface and thereby reduce the associated workload required to address frequent air leaks. Additionally, refined work flow processes could be investigated to find ways to further streamline the device set up process. Given the increased human resource demands of bCPAP, future research is needed to assess the cost-effectiveness of bCPAP compared to low flow oxygen.

Several factors limit the interpretation of these results. Observations were completed in the context of a clinical trial with a higher staff to patient ratio than would be usually present on a general pediatric hospital ward. Study staff were following a protocol which sought to balance treatment representative of real-world care provided in a district hospital with the rigors of a clinical trial. The protocol, therefore, may have added increased time and tasks over care provided outside of a study setting, but this was generally balanced equally between study groups. There is also the potential for the observer effect and observer drift across observations. We attempted to minimize observer drift through regular supervision and reinforcement of observation methods with observers. The same staff members were supervised daily and observers limited contact with staff during ongoing observations, which should decrease the influence of the observer effect over time. Overall, however, the randomized design of the parent study should account for unmeasured confounders, such that observed differences can be attributed to the form of respiratory support. Lastly, since we did not reach our targeted sample size due to premature closure of the trial, some comparisons may be underpowered and our results should be interpreted within this context.

## Conclusion

bCPAP takes significantly longer to initiate and maintain compared to the current standard of care of low-flow nasal oxygen and may therefore require healthcare workers to shift between tasks more frequently. Consequently, widespread implementation of bCPAP at the district hospital level in Malawi will likely require substantial additional human resources. If this is not considered, the quality of bCPAP care itself as well as care for all hospitalized patients is likely to be detrimentally affected.

## Additional files

Additional file 1: **Table S1.** Interobserver reliability assessment results for the two observers. Statistical comparisons assessing interobserver reliability between the two observers prior to the start of observations. (DOCX 15 kb)

Additional file 2: **Table S2.** Demographic information of children observed. Demographics of participants by group including age, gender, weight, distance to nearest health facility, HIV status, SAM, vitals on admission, Blantyre Coma Scale, and hemoglobin. (DOCX 15 kb)

Additional file 3: **Table S3.** Demographics of patients whose care was observed and all patients enrolled in CPAP IMPACT. Comparison of the subset of CPAP IMPACT patients observed to the entire study population including age, gender, weight, and SpO2. (DOCX 14 kb)

## Data Availability

The datasets used and/or analyzed during the current study are available from the corresponding author on reasonable request.

## Abbreviations

bCPAP: Bubble continuous positive airway pressure
CPAP IMPACT: Improving Mortality for Pneumonia in African Children Trial
HCW: Health care workers
HIV: Human immunodeficiency virus
IORA: Interobserver reliability assessment
PEEP: Positive end expiratory pressure
SD: Standard deviation
SDH: Salima District Hospital
TMS: Time motion study
WHO: World Health Organization
