# Supplementary material for: Bubble CPAP and oxygen for child pneumonia care in Malawi: a CPAP IMPACT time motion study
Source: BMC Health Serv Res. 2019 Jul 31;19:533. doi: 10.1186/s12913-019-4364-y (PMC6668155; doi:10.1186/s12913-019-4364-y)
Supplement: Supplementary file 1 — Table S1. Interobserver reliability assessment results for the two observers. Statistical comparisons assessing interobserver reliability between the two observers prior to the start of observations. (DOCX 15 kb) [file 12913_2019_4364_MOESM1_ESM.docx]

**Additional file 1: Table S1:** Interobserver reliability assessment results for the two observers

| Initiation | | | | | | | | | | | | | | | | | | | | |
| --- | --- | --- | --- | --- | --- | --- | --- | --- | --- | --- | --- | --- | --- | --- | --- | --- | --- | --- | --- | --- |
|  | Clinical Officer | | | | | | | Nurse | | | | | | HIV Counselors | | | | | | |
|  | P-K | | N-K | | D-CCC | S-NW | | P-K | | N-K | | D-CCC | S-NW | P-K | | N-K | | D-CCC | | S-NW |
| IORA 1 | 77.08%  0.52 K | | 48.72%  0.33K | | 0.87p | 0.65 | | 70.99%  0.53 K | | 46.43%  0.33 K | | 0.97 p | 0.61 | 91.13%  0.88 K | | 47.83%  0.32K | | 0.88 p | | 0.76 |
| IORA 2 | 96.47%  0.88 K | | 66.67%  0.57 K | | 1 p | 0.79 | | 87.52%  0.78 K | | 67.57%  0.62 K | | 0.95 p | 0.74 | 98.56 %  0.97 K | | 90.91%  0.85 K | | -0.26 p | | 0.82 |
| Maintenance | | | | | | | | | | | | | | | | | | | | |
|  | | Clinical Officer | | | | | | | | | Nurse | | | | | | | | | |
|  | | P-K | | N-K | | | D-CCC | | S-NW | | P-K | | | | N-K | | D-CCC | | S-NW | |
| IORA 1 | | 98.79%  0.94 K | | 28.57 %  0.24 K | | | 0 p | | 0.8 | | 93.43 %  0.76 K | | | | 43.75%  0.29K | | 0.99 p | | 0.81 | |
| IORA 2 | | 99.01 %  0.96 K | | 50%  0.31 K | | | 0 p | | 0.8 | | 89.44 %  0.75 K | | | | 63.16 %  0.55 K | | 1 p | | 0.76 | |

P-K indicates proportion-kappa; N-K, Naming PK; D-CCC, duration-concordance correlation coefficient; S-NY, sequence-Needleman-Wunsch; IORA, intraobserver reliability assessment.
