# Supplementary material for: Bubble CPAP and oxygen for child pneumonia care in Malawi: a CPAP IMPACT time motion study
Source: BMC Health Serv Res. 2019 Jul 31;19:533. doi: 10.1186/s12913-019-4364-y (PMC6668155; doi:10.1186/s12913-019-4364-y)
Supplement: Supplementary file 2 — Table S2. Demographic information of children observed. Demographics of participants by group including age, gender, weight, distance to nearest health facility, HIV status, SAM, vitals on admission, Blantyre Coma Scale, and hemoglobin. (DOCX 15 kb) [file 12913_2019_4364_MOESM2_ESM.docx]

**Additional file 2: Table S2:** Demographic information of children observed

|  | Total  (n=40) | bCPAP  (n = 19) | Oxygen  (n = 21) | p value * |
| --- | --- | --- | --- | --- |
| Age in months, mean (SD) | 11.35 (10.48) | 11.51 (12.05) | 11.21 (9.13) | 0.93 |
| Females, n (%) | 16 (40.00) | 8 (42.11) | 8 (38.10) | 0.80 |
| Weight in kg, mean (SD) | 7.39 (3.17) | 7.11 (3.64) | 7.64 (2.76) | 0.19 |
| Distance from nearest health facility (mean, SD) | 5.51 (6.83) | 7.06 (9.40) | 4.19 (3.06) | 0.40 |
| HIV Status, n (%) |  |  |  |  |
| Infected | 2 (5.00) | 1 (5.26) | 1 (4.76) | 0.94 |
| Exposed | 3 (7.50) | 0 (0) | 3 (14.29) | 0.09 |
| Severe Acute Malnutrition, n (%) | 15 (37.50) | 8 (42.11) | 7 (33.33) | 0.56 |
| Vitals on admission, mean (SD) |  |  |  |  |
| Respiratory rate | 65.80 (15.41) | 62.53 (18.02) | 68.76 (12.32) | 0.21 |
| Heart rate | 164.75 (25.97) | 163.68 (24.29) | 165.71 (27.96) | 0.81 |
| Oxygen saturation | 85.35 (10.70) | 86.11 (18.02) | 84.67 (7.16) | 0.74 |
| Temperature (°C) | 37.62 (1.29) | 37.14 (1.31) | 38.05 (1.13) | 0.02 |
| Blantyre Come Score <5, n (%) | 4 (10.00) | 2 (10.53) | 2 (9.52) | 0.92 |
| Hemoglobin in g/dL, mean (SD) | 9.86 (2.52) | 9.76 (2.79) | 9.95 (2.31) | 0.82 |

bCPAP indicates bubble continuous positive airway pressure; SD, standard deviation; HIV, human immunodeficiency virus.

*****p value comparing bCPAP and low-flow nasal oxygen cohorts, p<0.05 considered significant
