# Supplementary material for: Bubble CPAP and oxygen for child pneumonia care in Malawi: a CPAP IMPACT time motion study
Source: BMC Health Serv Res. 2019 Jul 31;19:533. doi: 10.1186/s12913-019-4364-y (PMC6668155; doi:10.1186/s12913-019-4364-y)
Supplement: Supplementary file 3 — Table S3. Demographics of patients whose care was observed and all patients enrolled in CPAP IMPACT. Comparison of the subset of CPAP IMPACT patients observed to the entire study population including age, gender, weight, and SpO2. (DOCX 14 kb) [file 12913_2019_4364_MOESM3_ESM.docx]

**Additional file 3: Table S3:** Demographics of patients whose care was observed and all patients enrolled in CPAP IMPACT

|  | TMS sample  (n=40) | CPAP IMPACT  (n = 644) | p value |
| --- | --- | --- | --- |
| Age in months, mean (SD) | 11.35 (10.48) | 11.84 (11.92) | 0.79 |
| Females, n (%) | 16 (40.00) | 345 (53.57) | 0.09 |
| Weight in kg, mean (SD) | 7.39 (3.17) | 7.16 (2.84) | 0.62 |
| SpO_2_, mean (SD) | 85.35 (10.70) | 86.21 (11.55) | 0.64 |

TMS indicates time motion study; CPAP IMPACT, Continuous Positive Airway Pressure Improving Mortality for Pneumonia in African Children Trial; SD, standard deviation; SpO_2_, peripheral oxygen saturation.
